# Psychophysiology of neural, cognitive and affective integration: fMRI and autonomic indicants

**DOI:** 10.1016/j.ijpsycho.2009.01.012

**Published:** 2009-08

**Authors:** Hugo D. Critchley

**Affiliations:** Brighton and Sussex Medical School Falmer Campus Brighton BN1 9RR UK

**Keywords:** fMRI, Central nervous system, Autonomic nervous system, Sympathetic, Parasympathetic, Cognitive, Affective

## Abstract

Behaviour is shaped by environmental challenge in the context of homoeostatic need. Emotional and cognitive processes evoke patterned changes in bodily state that may signal emotional state to others. This dynamic modulation of visceral state is neurally mediated by sympathetic and parasympathetic divisions of the autonomic nervous system. Moreover neural afferents convey representations of the internal state of the body back to the brain to further influence emotion and cognition.

Neuroimaging and lesion studies implicate specific regions of limbic forebrain in the behavioural generation of autonomic arousal states. Activity within these regions may predict emotion-specific autonomic response patterns within and between bodily organs, with implications for psychosomatic medicine. Feedback from the viscera is mapped hierarchically in the brain to influence efferent signals, and ultimately at the cortical level to engender and reinforce affective responses and subjective feeling states. Again neuroimaging and patient studies suggest discrete neural substrates for these representations, notably regions of insula and orbitofrontal cortex.

Individual differences in conscious access to these interoceptive representations predict differences in emotional experience, but equally the misperception of heightened arousal level may evoke changes in emotional behaviour through engagement of the same neural centres. Perturbation of feedback may impair emotional reactivity and, in the context of inflammatory states give rise to cognitive, affective and psychomotor expressions of illness. Changes in visceral state during emotion may be mirrored in the responses of others, permitting a corresponding representation in the observer. The degree to which individuals are susceptible to this ‘contagion’ predicts individual differences in questionnaire ratings of empathy. Together these neuroimaging and clinical studies highlight the dynamic relationship between mind and body and help identify neural substrates that may translate thoughts into autonomic arousal and bodily states into feelings that can be shared.

## 1 Introduction

Psychophysiological science is grounded on the premise that the mind is embodied: Mental processes influence the physiological state of the body while changes in the body's physiology influence thoughts, feelings and motivational behaviour. Homoeostasis is a pervasive guiding principle: Motivations arise from physiological need to preserve the integrity of the organism, via processes including thermoregulation, maintenance of fluid and nutrient balance and avoiding the consequences of noxious stimuli. Thus, the way we process or react to our environment depends on our internal bodily state. For example if we are thirsty or hungry, the manner in which we behave toward food stimuli differs and biases perceptions, hedonics, cognitions and memory. Motivations drive behaviour, while changes in the internal state of the body anticipate, facilitate or accommodate the consequences of motoric action. The result is a system (the individual) in a state of dynamic flux internally that contributed to physical and social interaction with the external environment.

As a consequence, salient events and emotive stimuli in our environment influence our internal bodily state. The automaticity of these physiological reactions, particularly to threat, suggests an independence of emotional reaction from ‘rational’ thoughts. The James–Lange theory of emotion proposes that emotional feelings arise from the mind perception of bodily changes in response to emotive stimuli to ‘colour’ our thoughts (James, 1894; Lange, 1885). Arguably obligatory changes in bodily response are viewed as definitive to emotion, and observed automaticity in bodily reactions is taken to indicate (precognitive) primacy. Implicit also to the James–Lange theory is the notion that different bodily states accompany different subjective emotions. Moreover, individual differences in the quality of bodily representation may underlie individual differences in emotional experience: Someone ‘constitutionally’ attuned to sensations from their heart may experience some emotions (perhaps fear and love) with greater intensity than another individual with a stronger representation of the functioning of the stomach. Nevertheless, arguments against the James–Lange theory put forward by Walter Cannon and others (Cannon, 1927) (e.g. bodily arousal responses too undifferentiated, no evidence of primacy) have led many psychologists to ascribe to a compromise view. Schachter and Singer (1962) proposed that a change in bodily arousal triggers and provides the intensity to emotion, which is then cognitively ascribed valence and quality according to the context; i.e. if we feel out heart race it is fear, not love, because we see a spider. However the underlying unidimensional model of bodily arousal state, though a widely held heuristic, fails to reflect the rich and subtle patterning of organ-specific responses regulated by the autonomic nervous system.

Interaction between bodily reactions and cognitive processes has remained toward the periphery of psychological science, and still all too often treated as a confound. Commonly a single metric of autonomic nervous function may be used as an objective index of mental processing, but the response itself is treated as an interesting epiphenomenon rather than integral to the cognition or emotion. Nevertheless, the functional impact of two-way communication between mind and body has long been recognized with implications for fields such as psychosomatic medicine. Damasio and colleagues reinvigorated the field with observations in brain lesioned patients and healthy individuals that led to the formulation of the Somatic Marker Hypothesis: Cognitive processes such as decision-making are guided by central feedback of bodily arousal responses (Damasio et al., 1991; Damasio, 1994, 1999). Ultimately a comprehensive understanding of neural, cognitive and affective processes must acknowledge the integration of mental and bodily processes. An integrative investigative approach is called for, combining techniques including functional brain imaging and detailed autonomic monitoring, and drawing on both animal studies and clinical observations.

The autonomic nervous system and accompanying visceral afferent fibres represent the principal neural channels through which the brain and internal bodily organs interact (e.g. Brading, 1999; Jänig, 2008). Sympathetic and parasympathetic divisions regulate ‘vegetative’ autoregulatory processes and responses engendered by dynamic interactions with the environment. Autonomic arousal is generally understood as a shift in visceral state to facilitate on-going or anticipated motor action, through an increase in cardiac output and blood flow to musculature and a parallel reduction in blood supply to the gut. This process is associated with a general increase in sympathetic activity particularly to heart, blood vessels and skin and is typically preceded by a more rapid withdrawal of parasympathetic activity. This ‘fight or flight’ pattern of bodily response is produced stereotypically to a range of perceived environmental threats that as a minimum capture attention. To lesser degrees the same pattern accompanies shifts in attention and cognitive evaluations with negative behavioural connotations. The unidimensional concept of physiological arousal, based on such observations, has widespread appeal and general validity particularly in models of emotion. However, it hides the detailed goal-orientated organ specificity apparent across both sympathetic and parasympathetic autonomic axes (Cannon, 1927; see also Porges, 1995; Morrison, 2001). A subset of autonomic arousal responses have developed into potent social cues that can betray an individual's motivational state (Darwin, 1989; Ekman et al., 1983).

A starting point for understanding cognitive, affective and autonomic integration is to define brain mechanisms that relate to the generation and feedback representation of bodily arousal responses during cognitive and affective behaviours. Functional magnetic resonance (fMRI) brain imaging enables measurement across whole brain of local haemodynamic changes that reflect local neural activity. The technique has reasonable spatial (a few millimetres) and temporal resolution (seconds), limited by the distribution and responsivity of cerebral arterioles. Within these constraints, fMRI has permitted some significant advances in understanding brain processes supporting cognition and emotion (Frackowiak et al., 2003). Combining these techniques with autonomic monitoring and studies of autonomic patients within my own research has permitted further insight into brain body interactions and the integration of autonomic affective and cognitive processes (Critchley and Dolan, 2003; Critchley, 2005).

## 2 Imaging autonomic interaction with cognition

From the outset functional neuroimaging of the brain has tended to reveal increased activity in dorsal anterior cingulate cortex when people are engaged in demanding tasks. Brain imaging experiments, particularly H_2_^15^O positron emission tomography (PET) studies, typically contrasted performance of a task containing cognitive/emotional process of interest with a control task that was similar in terms of sensorimotor performance requirements but lacked the ‘extra’ element. Radiolabelled water accumulated where there was more brain activity. Paus et al. (1998) reviewed 107 such studies all of which activated the same region of dorsal anterior cingulate cortex, which he ascribed to non-specific behavioural effort (largely resulting from task imbalance). Raichle and et al. (2001) similarly point to deactivation of the ventromedial prefrontal cortex and subgenual cingulate cortices in the same context.

**Fig. 1:**
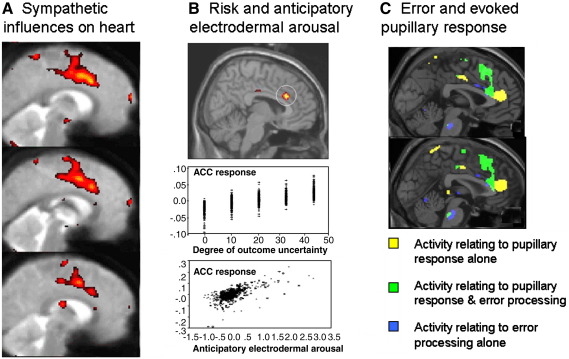
Functional magnetic resonance imaging (fMRI) studies revealing activity within mid and dorsal anterior cingulate cortex associated with integration of cognitive processes with changes in autonomic bodily states. A. Participants were scanned performing a working memory (n-back) task and an isometric exercise task at two levels of difficulty. Simultaneous electrocardiography was recorded throughout the study and post processed to drive a measure of sympathetic influences on heart rate (low frequency component of heart rate variability) in time. Group activity is shown on sagittal midline sections of an average echoplanar image, illustrating enhanced brain activity covarying with increasing sympathetic power, showing engagement of dorsal anterior and mid cingulate cortices (Critchley et al., 2003). B. Participants were scanned using fMRI during performance of a gambling task in which they saw a playing card and decided, to win money, if the next card would be higher or lower in face value. Electrodermal activity, reflecting sympathetic innervation of skin, was recorded and measures of anticipatory electrodermal arousal were derived for the time period between the cards i.e. before outcome. Activity within a region of dorsal anterior cingulate cortex is shown in the top panel plotted on a section from a standard normalized anatomical image. Beneath are the correlations of this activity with increasing risk in the decision just taken, and with anticipatory electrodermal arousal. The same brain region was found to mediate both (Critchley et al., 2001a,b). C. Activity changes within the dorsal anterior cingulate cortex reflecting aspects of Stroop task performance within fMRI, conducted with simultaneous pupillometry. Differences in the light reflex (to isometric stimuli) were derived in an event-related way, the largest effect (blunting of pupil constriction) occurring on error trials. The figure shows the location of enhanced activity relating to pupil size (yellow) on non-error trials, pupil size on error trials (green) and errors independent of pupillary change (Critchley et al., 2005a,b). Group data for different effects are plotted for illustration on sagittal sections of a standard anatomical image. The key is given in these panels. (For interpretation of the references to colour in this figure legend, the reader is referred to the web version of this article.)

Using first PET and later fMRI, my research within R. Dolan's laboratory, and the research of others has highlighted the close relationship between dorsal anterior cingulate activity and enhancement of autonomic arousal. In the first set of studies, healthy subjects performed mental arithmetic (serial subtractions) and a motor exercise task (isometric handgrip). Dorsal anterior cingulate activity was enhanced when performing the effortful versions of each of these tasks. More conclusively, activity within the dorsal anterior cingulate cortex correlated with increases in blood pressure induced (sympathetically) by mental or physical effort (i.e. independent of task) (Critchley et al., 2000). These findings were replicated in a study of older subjects (Critchley et al., 2001a). An adapted version of the same task was undertaken using fMRI. Here the sympathetic component of heart rate variability was measured while subjects performed working memory and isometric exercise tasks. Again activity in dorsal anterior/mid cingulate cortex correlated with sympathetic cardiovascular influences (Critchley et al., 2003) (Fig. 1A). These findings suggested dorsal anterior cingulate cortex to be important in the generation of autonomic arousal accompanying cognitive and volitional behaviour, a view endorsed by observations in patients with peripheral autonomic failure and three subjects with lesions to anterior cingulate cortex (Critchley et al., 2001a, 2003; Critchley, 2005).

Electrodermal activity is an interesting autonomic measure to psychophysiology in that it reflects sympathetic neural responses independent of direct parasympathetic control or circulating factors such as adrenaline. Evoked electrodermal responses were central to the formulation of the Somatic Marker Hypothesis by Damasio and co-workers. Lesion and stimulation studies have particularly helped map the brain regulation of this pure sympathetic index of cognitive-autonomic integration. One such study highlighted the hierarchy of descending electrodermal control (Mangina and Beuzeron-Mangina, 1996). In human subjects (adult surgical patients), direct electrical stimulation of amygdala, hippocampus and anterior cingulate regions strongly modulated electrodermal responses ipsilaterally, whereas stimulation of the frontal neocortex and mid region of the second temporal gyrus produced absent or weak ipsilateral, contralateral or bilateral responses.

In a set of neuroimaging studies, following on from the work of Damasio's group, we examined the brain control and representation of autonomic arousal responses (using electrodermal activity) during motivational decision-making; i.e. gambling task performance. In particular, one such study revealed activity within the dorsal anterior cingulate cortex in anticipation of the outcome of risky decisions that reflected both the degree of risk in the decision and the electrodermal arousal in anticipation of the outcome of the decision (a judgment as to whether a playing card would be followed by a higher or lower card) (Critchley et al., 2001b) (Fig. 1B). A similar observation in a ‘wheel of fortune’ study indicated the importance of agency in the decision-making process. Despite outcomes having the same monetary value, if a participant *actively* selected which gamble they wanted to play, heart rate increased, both in anticipation of the outcome and in response to the feedback given at outcome. Notably the genual and dorsal anterior cingulate cortex was engaged at outcome reflecting both the agency and the autonomic state accompanying that agency (Coricelli et al., 2005).

A further example of the integration of brain and autonomic response comes from a study of the Stroop interference task. Dorsal anterior cingulate cortex has been strongly implicated in executive function. A functional system centred around the dorsal ACC for rapid detection and signalling of cognitive and behavioural errors was identified using depth electrodes in the human brain (Bechtereva, 1987; Bechtereva et al., 1990, 2005). Such findings have been extended and reinforced by electrophysiological and neuroimaging data that highlight dorsal ACC engagement during cognitive conflict and error, indirectly via midline cortical potentials or within changes in haemodynamic responses (e.g. Dehaene et al., 1994; Bush et al., 2000). Within the Stroop task, cognitive conflict is generated where a response is required that goes against a prepotent or ‘more natural’ response to one of sensory attributes of the stimuli. For example, we conducted a study using a numerical Stroop task, in which the numerical and physical size of number stimuli was modulated to generate to congruent and incongruent trials (where physical and numerical size did not match). In our study, we also measured autonomic arousal responses from changes in pupil size in response to individual Stroop trials and correlated this with stimulus evoked brain activity. Behaviourally, errors in Stroop task performance elicited the greatest variance in pupil size, reflecting the trend toward larger pupils (increased sympathetic response) when a subject made an error (always on incongruent trials) (Critchley et al., 2005a). Corresponding brain activity showed a region of medial cortex including dorsal anterior cingulate and pre supplementary motor area, that mapped both performance error and associated pupillary arousal response (Fig. 1C). Prior EEG studies have associated the occurrence of autonomic responses not so much with conflict processing, nor even with the ‘fast’ error related negativity potential, but with an error related positivity that is associated with conscious awareness of having made an error (Nieuwenhuis et al., 2001; Hajcak et al., 2003). Our study suggests a common substrate within dorsal ACC for these aspects of error processing.

Together these studies link autonomic arousal to decision-making anticipation of outcome and processing outcomes beyond effort and point to the anterior cingulate cortex as a mediator of changes in sympathetic arousal coupled to effortful and evaluative processing. While low-level autonomic explanations account in part for task-related activity during cognitive behaviour; dorsal anterior cingulate engagement appears in the context of attentional engagement and volitional task performance that engender peripheral physiological changes. These effects are apparent across different autonomic axes, yet the different locations of concurrent cingulate cortical activity appear to reflect both task differences and the particular autonomic response that was measured.

## 3 Imaging autonomic interaction with affect

Anterior cingulate activity is further associated with generation of sympathetic autonomic responses in the context of emotional processing; again there appears to be some relation to the nature of the task and with the precise autonomic response measured. One study examined the effects of emotional processing on the cardiac responses induced during a forewarned reaction time task. Forewarned reaction time tasks induce predictable changes in both brain activity and autonomic bodily response. A warning stimulus (cue) signals that the participant should respond to the next imperative stimulus with a reaction time response. In the period between cue and imperative stimuli, there is an orienting response to the cue stimulus followed by cardiac deceleration prior to the imperative stimulus and response. An electrocortical potential (the contingent negative variation, CNV) also occurs during this anticipatory interval, indicative of thalamocortical excitability and ascribable in part to dorsal cingulate activation (e.g. Nagai et al., 2004). The emotional version of the forewarned reaction time task replaced the imperative stimuli with faces depicting different emotional expressions. The reaction time response was a choice judgement of the emotion portrayed. By measuring cardiac responses to the face stimuli it was possible to examine brain activity associated with differential cardiac responses (orienting acceleration and deceleration) to different emotional expressions. In fact the accelerative and decelerative cardiac responses were closely correlated. Processing of happy and disgust faces attenuated, while processing sad and angry faces enhanced, heart rate responses. Brain activity reflecting emotional effects on heart rate within and between the different categories of emotional processing was enhanced in regions including anterior cingulate cortex, amygdala and temporal lobe (lingual and fusiform) cortices (Critchley et al., 2005b).

The emotional effect observed on heart rate was consistent with those observed in other contexts: notably Ekman et al.'s studies on differential autonomic responses to emotion (Ekman et al., 1983). The basic principle; greater parasympathetic and perhaps less sympathetic activation associated with happiness and disgust and a shift toward sympathetic dominance and parasympathetic activity with sadness and anger, is replicated in other contexts and to other stimulus sets (e.g. Umeda et al., unpublished observations). These studies highlight a degree of emotion-specificity of autonomic response that is nevertheless crude compared to the organ-specific control visible as changes in posture, skin perfusion that characterise the expression of different emotions for which autonomic patterning is the rule.

## 4 Integration with afferent feedback

The internal state of the body influences the way we think and feel. Primary or basic emotions originate in states of physiological imbalance, including inflammation and pain. The physiological readiness of the body shapes the execution of particular behaviours linking cognitive and emotional processes to more dynamic changes in bodily state. The influence of transient arousal responses on aspects of affect and cognition is embodied within Damasio's Somatic Marker Hypothesis which proposed that emotional feelings originate in representations particular within somatosensory cortices (Damasio et al., 1991; Damasio, 1994, 1999). Empirical studies have refined these concepts and implicate insula cortex as the substrate for emotional feelings, supported by activity within amygdala, anterior cingulate cortex and orbital regions. One approach to link internal visceral processes to emotional feelings involves testing for individual differences in interoceptive awareness, particularly sensitivity to the beating of one's own heart. The logic originates in James–Lange and related peripheral theories of emotion: If internal physiological processes ultimately drive emotional feelings then differences in emotional experience and behaviour across individuals may be accounted for by differences in the degree to which people are constitutionally attuned to their own bodily processes.

An imaging study using a heart beat detection task revealed engagement of anterior cingulate insula and somatosensory cortices when people focus on their internal bodily processes. Awareness and sensitivity to the occurrence of individual heart beats was revealed by how well people distinguished the presence of a heart-note delay in auditory tones triggered by individual heart beats. Across participants, activity and even the grey matter volume within one brain region, right anterior insular cortex, predicted differences in interoceptive sensitivity (Critchley et al., 2004). The link with emotional process was revealed by showing that interoceptive sensitivity and the activity/volume within right insula cortex also reflected day to day experiences of anxiety (and to a lesser extent other negative emotions). This study reinforced proposals put forward by Craig that the right anterior insula cortex is the terminus of afferent spinal information from unmyelinated viscerosensory fibres. Since the information mapped by this system was essentially of motivational significance, Craig proposed that the insula cortex represents motivational state and through re-representations becomes the substrate for conscious feeling states (Craig, 2002, 2003).

## 5 Perturbation of visceral afferent information

Both visceral stimulation and general perturbations in interoceptive state modulate activity within similar brain centres. Direct gastrointestinal stimulation of oesophagus or large bowel enhances activity with cingulate and insula cortex (even in the absence of pain) (Hobday et al., 2001). Experimentally-induced inflammation using typhoid vaccine also modulates cortical and subcortical activities to predict changes in psychological and psychomotor function. For example, in a study by Brydon et al. (2008), experimental inflammation modulated activity within the substantia nigra (without affecting neurovascular coupling). Changes in this brain region were correlated with circulating cytokine IL6 and also predicted a general slowing of reaction time to task stimuli. Together these observations highlight multiple levels of interaction between afferent visceral information about perturbation in bodily state and cognitive motivational and general psychomotor processes.

Observations in patients with spinal cord lesions and from a single patient treated with vagus nerve stimulation for depression also highlight a role of ventromedial prefrontal cortex in the integration of afferent visceral sensory information perturbed by interference with spinal and vagus nerve routes (Nicotra et al., 2006; Critchley et al., 2007). Surprisingly there does not seem to be an obvious functional hierarchy in these effects, something that other data also suggest. The conclusion here is that anterior insula and ventromedial prefrontal cortex contribute to the integration of visceral afferent information, generated by salient stimuli with stimulus processing itself.

## 6 Phasic visceral influences on stimulus processing

Afferent information from the viscera influences stimulus processing even at the level of individual heart beats (Lacey and Lacey 1978, 1980). At systole the pressure wave of cardiac ejection activates aortic and carotid baroreceptors. Baroreceptor effects influence the processing of painful and strong unexpected somatosensory stimuli to modify cortical reflexive and autonomic response (Edwards et al., 2003; Donadio et al., 2002; Wallin, 2007). A link to cognition is also apparent in studies examining cardiac cycle influences on timing tasks (Somsen et al., 2004). Thus efferent sympathetic responses to stimuli occurring at systole are modulated notably there is inhibition of muscle sympathetic nerve traffic while sympathetic electrodermal responses are unaltered. These effects are amplified in patients with blood phobia and syncope, suggesting that these mechanisms are central to traits in emotional behaviour and reactivity (Donadio et al., 2007). Our own neuroimaging observations (Gray et al., 2009) reveal that the interaction between stimuli and baroreceptor activation within the cardiac cycle is mediated by differential engagement of a discreet set of brainstem, cortical and subcortical centres to elicit further characteristic changes in autonomic state.

## 7 Cognitive influences on emotional processing mediated through interoceptive representation

**Fig. 2:**
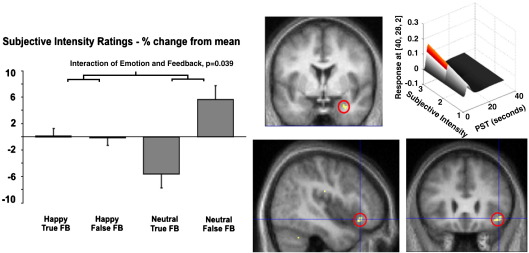
Cognitive influences on emotional processing mediated through interoceptive representation. Participants were scanned using fMRI while they performed judgments of faces depicting emotional and neutral expressions. During the task they also performed blocks of handgrip exercise (and no exercise). Throughout the study they were played through headphone auditory tones that they were led to believe reflected their immediate heart rate. In fact this feedback was either valid or invalid (asynchronous or accelerated). When participants heard accelerated ‘heart sounds’ when not exercising, they rated neutral faces to be more emotionally salient. False feedback was not found to influence ratings of strongly emotional faces and states of true arousal attributable to exercise did not influence neutral or emotional face judgment. The change in ratings to neutral faces is illustrated in the left panel. Brain activity (right panels) within amygdala and anterior insula reflected the interaction between false feedback and neutral emotional judgment, and correlated with the change in subjective rating for neutral faces (top right panel for amygdala). Group data are plotted on orthogonal sections of a normalized echoplanar image (see Gray et al., 2007).

The representation of afferent information from the viscera and to emerge as consciously accessible emotional feelings appears to be supported at a neural level through the activity of right anterior insula cortex. In fact the perception of internal state of arousal may be influenced but false feedback. In an imaging study, participants were fed back auditory tones which they were led to believe represented their heart beating. During the course of the experiment they were required to judge the emotional ‘intensity’ of neutral happy and angry faces while undertaking or not undertaking blocks of isometric handgrip exercise. Behaviourally, the participants rated neutral (but not intrinsically emotional) faces as more intense/salient when they heard a heart beat trace consistent with arousal that would normally accompany exercise, but were not exercising. The change in rating of neutral faces due to false feedback arousal was reflected in activity changes in the right anterior insula and amygdala (Gray et al., 2007). These observations fit with notions of ‘excitation transfer’ and attribution of arousal. During exercise the perceived change in autonomic state is attributable to its proximate cause, exercise, and causes negligible effect on ratings of emotional and neutral faces. Similarly during false feedback, the perception of ‘extra arousal’ is seemingly transferred and attributed to neutral stimuli with low intrinsic arousal to alter judgment about the emotional value of these stimuli, but does not significantly influence processing of strongly emotional stimuli that carry intrinsic arousal that may be viewed as intrinsically arousing (Fig. 2). These observations also map into the insula theory of anxiety of Paulus and Stein (2006) that proposes feelings of anxiety emerge through mismatched representation of anticipated and perceived bodily state within the insula cortex.

## 8 Autonomic communication and empathy

The neural integration of cognitive, affective and autonomic response has been proposed to be a guide to adaptive social behaviour (Damasio et al., 1991; Damasio, 1994, 1999). Changes in visceral state during emotion may be mirrored in the responses of others, permitting a corresponding representation in the observer. The degree to which individuals are susceptible to this ‘contagion’ predicts individual differences in questionnaire ratings of empathy. The notion that affective feelings are coupled to autonomically-mediated visceral responses implies that the sharing of emotional feelings empathetically embodies a sharing of visceral autonomic response across individuals. A dependence of emotional interchange on aspects of autonomic reactivity is suggested from observations in patients with primary autonomic failure, who manifest a blunting of empathetic responses on a questionnaire measure of emotional empathy (Chauhan et al., 2008). With many autonomic responses serving as visual signals of emotion, reciprocation is visibly evident in the contagion of fear (including facial pallor) and anger responses (facial flushing), but in many cases the exchange is subtle and the signals covert.

**Fig. 3:**
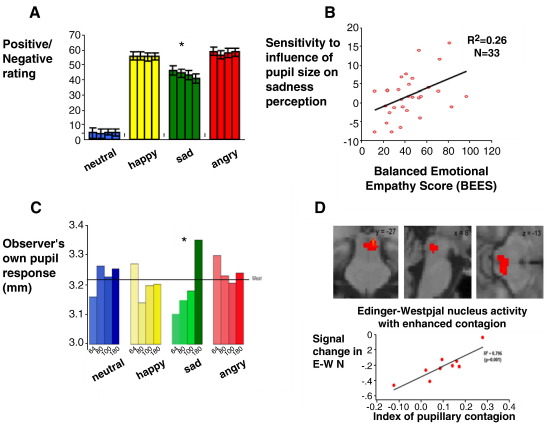
Autonomic mirroring in pupil responses to sad faces. A. When judging emotional expressions, pupil size biases the perception of sad facial expressions. When students rate pictures of faces, unaware of manipulations to pupil size across images, they rate sad facial expressions as more intense and negative when the observed pupil size is smaller (Harrison et al., 2006). For each emotion the four bars represent from left to right increasing pupil size on the ratted images (from 64% of the actual size to 180%). B. The degree to which individuals show that this effect correlates with questionnaire ratings of emotional empathy (Harrison et al., 2007). C. A neuroimaging study was conducted with simultaneous pupillometry. When participants judge the age of emotional faces, pupil manipulations are mirrored in their own pupils as responses (recovery of light reflex); seeing small pupils on sad faces results in greater pupil constriction. Again for each emotion the four bars represent from left to right increasing pupil size depicted on the rated images (from 64% of the actual size to 180%). Within individuals, this effect was associated with changes in activity within amygdala, operculum, superior temporal cortex and midbrain, in the region of the Edinger–Westphal nucleus (Harrison et al., 2006). D. Midbrain activity in the region of the Edinger–Westphal nucleus correlated across individuals with the degree to which they demonstrate pupillary contagion (in the context of sad faces). The parameter estimates of the size of the effect is plotted beneath individual differences in Edinger–Westphal nucleus activity plotted on sagittal and coronal sections of a normalized scan (Harrison et al., 2006).

One set of studies examined the impact of pupillary signals on emotional processing (Harrison et al., 2006, 2007; see Fig. 3). Student volunteers were asked in a behavioural task to make visual analogue ratings of positive/negative, intensity and attractiveness attributes of pictures of emotional faces. For each identity/expression combination, the size of the stimuli pupils was digitally manipulated to cover a range of (biologically plausible) sizes. Participants were not informed of this image manipulation and none was aware of this at debriefing after the task was performed. Contrary to the initial prediction that enlarged pupils, reflecting sympathetic activity, would produce greater intensity ratings of all the emotions, no significant (linear) effect of pupil size was observed for any of the emotions except sadness. Here the smaller the pupils the more negative and more emotionally intense sad faces were rated. Interestingly, the degree to which participants were affected by this covert manipulation of perceived pupil size correlated with individual differences in questionnaire measures of emotional empathy. A parallel neuroimaging study was conducted with the same pupil-manipulated face stimuli. Participants made age judgments of the faces, processing both emotion and the manipulated pupil size incidentally. During scanning with fMRI, it was also possible to use pupillometry to record the pupillary response of the participant to the stimuli. Activity changes in amygdala, insula and superior temporal sulcus and brainstem (in the region of the Edinger–Westphal nucleus) reflected the interaction between perceived pupil size and sad versus non-sad emotion. When the pupil responses of the participants were examined, it was found that there was coherence in the observed and observer's pupils only in the context of sadness (i.e. seeing a sad face with small pupils caused the participants' own pupils to constrict more). The extent to which this occurred was again predicted by activity within the Edinger–Westphal nucleus (the autonomic nucleus responsible for pupillary control.

These observations show a contagion of autonomic responses in emotional processing, highlighting automatic mirroring only in the context of sadness perception that influences the judgment of sadness in a manner related to individual differences in empathy for others. Moreover the findings highlight the presence of organ specificity in emotional autonomic responses that are integrated with cognitive and emotional aspects of empathy within a discrete neural system.

## 9 Conclusions

Together these neuroimaging and clinical studies highlight the dynamic relationship between mind and body and help identify neural substrates that may translate thoughts into autonomic arousal and bodily states into feelings that can be shared. By combining functional MRI with autonomic monitoring during performance of cognitive and emotional tasks one can tease apart the mechanisms of interaction that arise from an embodied mind. Certain patterns to the neural integration of affect cognition and visceral response are evident. Firstly, the notion that autonomic processes and control is confined to brainstem must be dismissed. Secondly a discrete set of cortical brain regions, including anterior cingulate and anterior insula orchestrates the response and representation of bodily states in specific behavioural contexts. The midline ‘generator’ system is likely to interact closely with the lateral ‘representational’ system (Craig, 2003; Critchley, 2005). Nuclei within dorsal pons, often in conjunction with dopaminergic midbrain centre also appear critical in this integration, as shown in studies of phasic and tonic (Critchley et al., 2001a; Critchley, 2005) autonomic arousal responses. The amygdala contributes in both efferent and afferent representational levels of affective autonomic response and there is evidence for a contribution to declarative judgment of emotional salience.

Despite anatomical evidence for hierarchical organization of this system, low-level autonomic changes can impact directly on high-level cognitive functions, and at the same time cognitive representation of anticipated or misperceived arousal can impact on peripheral and early stages of emotional response. Together these neuroimaging and clinical studies highlight the dynamic relationship between mind and body and help identify neural substrates that may translate thoughts into autonomic arousal and bodily states into feelings that can be shared. The extension of these findings to detail the fundamental psychophysiological processes and basis for differences in phenotypical expression across individual is anticipated to lead to novel interventions that will have real impact in the field of psychological and psychosomatic medicine.
